# RGD targeting of human ferritin iron-oxide nanoparticles enhances in vivo molecular MRI of experimental aortic aneurysms

**DOI:** 10.1186/1532-429X-14-S1-M9

**Published:** 2012-02-01

**Authors:** Toshiro Kitagawa, Hisanori Kosuge, Masaki Uchida, Yasunori Iida, Ronald L Dalman, Trevor Douglas, Michael V McConnell

**Affiliations:** 1 Cardiovascular Medicine, Stanford University School of Medicine, Stanford, CA, USA; 2 Chemistry and Biochemistry, Montana State University, Bozeman, MT, USA; 3 Vascular Surgery, Stanford University School of Medicine, Stanford, CA, USA

## Background

Both inflammation and angiogenesis contribute to the progression of abdominal aortic aneurysm (AAA) disease. RGD is a peptide binder of the αvβ3 integrin, which is expressed highly on activated macrophages and angiogenic endothelial cells. Human ferritin (HFn) is a nanoscale protein cage with 12nm diameter and 8nm interior cavity, which we have utilized as a platform for molecular/cellular imaging. We can genetically introduce RGD peptide to HFn. The purpose of this study is to evaluate RGD-conjugated HFn iron oxide nanoparticles for enhanced *in vivo* MRI of murine AAAs.

## Methods

1) *Mice* - Murine AAAs were induced in Apo-E deficient mice by angiotensin II infusion (1µg/kg/min), followed by monitoring of aortic diameter by ultrasound. Control mice were created by saline infusion.

2) *RGD-conjugated HFn-iron oxide nanoparticles* - HFn was genetically engineered to display 24 copies of an RGD peptide on the exterior surface of the protein cage. Magnetite (Fe_3_O_4_) was encapsulated in the interior cavity of RGD-conjugated HFn (RGD^+^) and non-targeted HFn (RGD^-^) at loading factors of 5000Fe per cage, giving R2 values of 93 mM^-1^s^-1^ (magnetite diameter: 5-7nm). The injected dose was adjusted to 25mgFe/kg in each animal.

3) *MRI* - All mice were imaged on a whole-body 3T MRI scanner (Signa HDx, GE Healthcare) with a phased array mouse coil (RAPID MR International), using a gradient echo sequence (TR/TE=100ms/10ms, slice thickness=1.0mm, FOV=3cm, matrix=256x256, FA=60, NEX=10). Mice were then injected with either RGD^+^ or RGD^-^ (6 AAA and 4 control mice for each), followed by MRI at 24 and 48 hours post injection. The nanoparticle accumulation was assessed by measuring the reduction in the T2*-weighted signal intensity of the AAA (or suprarenal aorta in control mice) relative to adjacent normal-size aorta (expressed as % SI loss).

4) *Histology* - The aortic wall was stained with Perl’s iron (for nanoparticle accumulation), CD-11b (for macrophages), and CD-31 (for endothelial cells).

## Results

**Figure 1:**
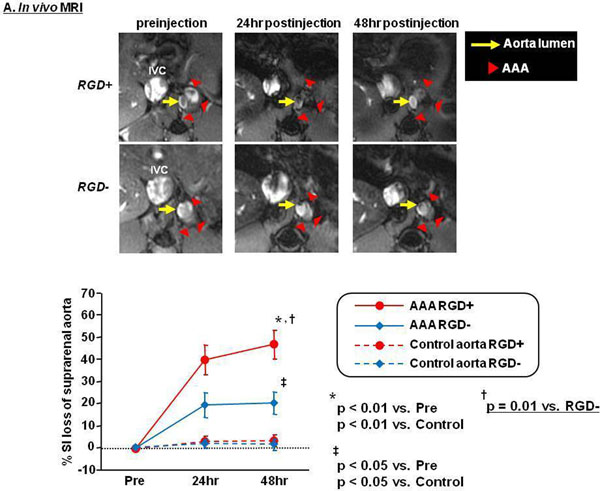


**Figure 2:**
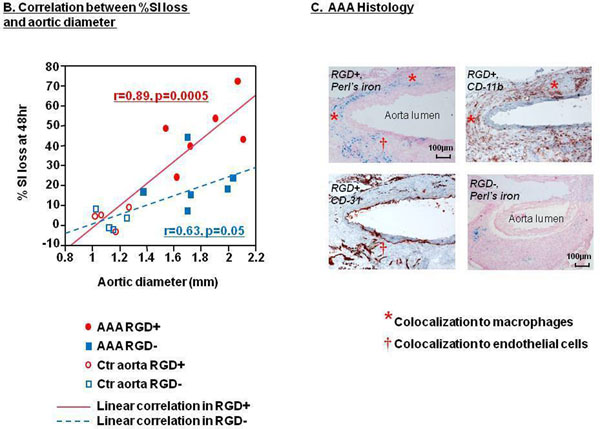


MRI showed greater T2^*^ signal loss in the AAA with RDG^+^ than with RDG^-^ (Fig 1(A)), confirmed by quantitative analysis of % SI loss (Fig 1(A) graph, p=0.01). Abdominal aortic diameter on ultrasound correlated more strongly with % SI loss with RDG^+^ than with RDG^-^ (Fig 2(B)). Perl’s iron staining confirmed greater accumulation of RDG^+^ in the AAA compared to RGD^-^ (474±51 vs. 277±29 stained cells/cross-sectional area, p=0.01), with colocalization to both macrophages (CD-11b) and endothelial cells (CD-31) within the AAA wall (Fig 2(C)).

## Conclusions

HFn iron-oxide nanoparticles with RGD targeting provide a promising MRI approach for comprehensive *in vivo* detection of inflammation and angiogenesis in high-risk AAAs.

## Funding

Dr. McConnell receives research support from GE Healthcare and he is on a scientific advisory board for Kowa, Inc.

